# Real-World Analysis of Organ Transplantation-Specific Agent Based on Large Language Model in Post-Transplant Self-Management During Off-Hours: A Mixed-Methods Study

**DOI:** 10.1007/s11596-026-00219-3

**Published:** 2026-07-01

**Authors:** Cheng Zeng, Xin Zhou, Hong-yun Xu, Xiao-ping Zhu, Yue Shi, Guang-le Dai, Zhi-gao Deng, Yan Xu, Li Xu, Hui Xiao, Shao-jun Ye

**Affiliations:** 1 Institute of Hepatobiliary Diseases of Wuhan University, Transplant Center of Wuhan University, National Quality Control Center for Donated Organ Procurement, Hubei Key Laboratory of Medical Technology on Transplantation, Hubei Provincial Clinical Research Center for Natural Polymer Biological Liver, Zhongnan Hospital of Wuhan University, Wuhan, 430071 China; 2 Party Committee Office, Zhongnan Hospital of Wuhan University, Wuhan, 430071 China; 3 Outpatient Department, Zhongnan Hospital of Wuhan University, Wuhan, 430071 China; 4 Information Center, Zhongnan Hospital of Wuhan University, Wuhan, 430071 China

**Keywords:** Organ transplantation, Large language models (LLM), Out-of-hours care, Artificial intelligence (AI), Patient self-management, Graph-based retrieval-augmented generation (GraphRAG), Mixed-methods study

## Abstract

**Objective:**

A significant gap exists in medical support for organ transplant patients during out-of-hours (OOH). General large language models (LLMs), affected by AI hallucinations, are unsuitable for complex post-transplant care. We built the first post-transplant AI agent based on LLMs to address these issues.

**Methods:**

We constructed a specialized “post-transplant AI agent” (named Doctor Xiao Yi) and conducted a mixed-methods study comparing it to a hospital-wide general AI agent (named Nan Xiao Yi). Data included 20,176 real-world logs (June–December 2025) and a cross-sectional survey of 152 transplant patients. We examined patterns of use over time, the types of questions raised, and the factors influencing patient behavior.

**Results:**

Unlike Nan Xiao Yi, Doctor Xiao Yi remained active during OOH, with a peak at 4:00 AM (*P* < 0.001). The general agent handled admin tasks like appointments, while the specialist agent provided clinical support such as diet, symptoms, and medication. Survey found 60.5% of OOH use by transplant patients due to reluctance to disturb human doctors. Furthermore, 63.8% of transplant patients were satisfied with the specialist agent's responses, and 48% reported they would decide on further hospital treatment based on AI suggestions.

**Conclusions:**

The specialist AI agent effectively fills the gap in medical and psychological services during OOH for transplant recipients. Based on the “dual-source knowledge base + GraphRAG + multi-agent framework” architecture, our specialist AI agent offers safe, reliable post-transplant care.

**Supplementary Information:**

The online version contains supplementary material available at 10.1007/s11596-026-00219-3.

## Introduction

Organ transplantation is a well-established and life-prolonging treatment for patients with end-stage organ failure. However, successful surgery marks only the beginning of long-term recovery. During the postoperative period, transplant recipients often experience a range of persistent physiological and psychological challenges. Clinical experience has shown that after hospital discharge, many patients report symptoms such as sleep disturbances, unstable blood pressure, and nocturia. Although these conditions rarely require urgent medical intervention, they can substantially impair patients’ quality of life. Concurrently, the psychological burden is profound; approximately one-third of recipients grapple with depression or anxiety, or even with posttraumatic stress disorder (PTSD). This convergence of somatic and psychic distress renders patients in a state of extreme vulnerability [1].

For patients recovering in a home-based setting, this vulnerability is exacerbated during out-of-hours (OOH) periods (late nights, early mornings, and holidays). When patients confront physical discomfort during these times, they often experience an “authority-dependent decision-making dilemma” [2, 3]. Owing to a deficit in specialized medical knowledge, immediate guidance from medical authorities is urgently needed to mitigate anxiety. However, traditional healthcare channels exhibit inherent limitations: emergency department visits entail arduous logistical processes, while immediate access to attending physicians via internet platforms is often unfeasible. This structural absence of medical support amplifies patient helplessness and increases the risk of adverse outcomes due to delayed management or improper handling [1].

In recent years, advances in large language models (LLMs) have attracted increasing attention in the medical field. These models have been explored for a range of applications, including medical examinations, clinical text summarization, and clinical decision support [4]. Nevertheless, the extant research has focused predominantly on evaluating general-purpose models (e.g., ChatGPT) within the context of generic medical knowledge or broad domains such as radiology. Evidence suggests that when complex clinical scenarios are navigated, generalist models are prone to “hallucinations”, lack domain-specific depth, and exhibit insufficient empathy, rendering them inadequate as direct substitutes for specialized practitioners [4–6]. To date, there is a paucity of literature reporting on the construction of an “AI specialist agent” specifically engineered for the nuanced postoperative management of solid organ transplantation.

To bridge this critical gap, this study constructed the inaugural “post-transplant AI agent” (named Doctor Xiao Yi) designed to provide 24/7 continuous medical support. Adopting a mixed-methods analysis approach, we conducted a comparative evaluation between this specialist agent and a hospital-wide general AI assistant (named Nan Xiao Yi). By rigorously analyzing 20,176 real-world interaction logs and 152 transplant patients’ feedback questionnaires, this study aims to evaluate the efficacy of the specialist agent in delivering dual medical-psychological support to transplant recipients during OOH periods and to explore its potential in providing data-driven support for the optimization of hospital resource allocation.

## Materials and Methods

### Study Design and Ethical Considerations

This study employed a mixed-methods research design, integrating a retrospective analysis of real-world interaction logs with a cross-sectional patient survey. The study protocol was reviewed and approved by the Ethics Committee of Zhongnan Hospital of Wuhan University (China). Informed consent was obtained electronically from all survey participants. For the retrospective analysis of conversation logs, strict data anonymization protocols were implemented to strip all personally identifiable information (PII), ensuring patient privacy and data security.

### Intelligent Agent Architecture: The “Doctor Xiao Yi” Framework

The specialist agent (Doctor Xiao Yi) was developed using a LLM (Tongyi Qianwen, China) framework fine-tuned for the organ transplantation domain, with an architectural focus on medical accuracy and emotional support.

Doctor Xiao Yi adopts a quad-dimensional technical architecture, as described below.

#### Dual-encapsulated authoritative knowledge base

Unlike general-purpose AI models trained primarily on open-web corpora, the specialist agent is grounded in a proprietary dual-source knowledge base. This includes (1) national-level clinical guidelines and expert consensus documents related to organ transplantation and (2) more than ten years of longitudinal follow-up data and accumulated clinical practice experience from our transplant center. This design ensures that all generated medical recommendations are evidence-based, context-aware, and clinically actionable. To maintain accuracy and currency, a “human-in-the-loop” update mechanism was established. A multidisciplinary committee of senior clinicians and clinical pharmacists periodically reviews and verifies new clinical guidelines and high-frequency unanswered patient queries before integrating them into the proprietary knowledge base.

#### GraphRAG with multipath recall

To mitigate the hallucinations inherent in generative models, a graph-based retrieval-augmented generation (GraphRAG) framework was implemented. The system integrates semantic vector retrieval with keyword-based search to enable multipath knowledge recall. This hybrid strategy provides millisecond-level precision in evidence matching and constrains model outputs to verified and traceable medical sources.

#### Multi-agent synergy with chain-of-thought reasoning

The specialist agent employs a multi-agent collaboration mechanism that simulates multidisciplinary expert consultation. Chain-of-thought (CoT) reasoning is used to replicate structured diagnostic and decision-making pathways like those followed by human specialists. In parallel, a dedicated intent recognition agent continuously analyzes user inputs to detect emotional cues and latent psychological needs, enabling the delivery of empathetic support alongside professional medical guidance.

#### Dynamic safety and continuous evolution mechanism

A multilevel risk defense network (MLRDN) is used to monitor interactions in real time. In high-risk scenarios, such as potential medical emergencies (e.g., self-reported temperature > 38.5 °C, black stool, and sudden chest pain), a circuit-breaker mechanism is automatically activated to redirect users toward immediate offline medical care (Supplementary File 1). To ensure medication safety, a mandatory risk-interception mechanism is implemented for immunosuppressants (e.g., tacrolimus); when queries involve dosage adjustments or drug combinations, AI is strictly prohibited from providing direct recommendations and instead outputs a standardized warning to consult a human physician or pharmacist. In addition, an automated evaluation pipeline based on the RAG assessment (RAGAS) framework continuously analyzes interaction data to identify unmet patient needs, supporting iterative optimization and ongoing evolution of the knowledge base.

In contrast, the general AI agent (Nan Xiao Yi) served as the control condition. It functioned as a standard hospital service assistant without domain-specific fine-tuning and was primarily responsible for administrative and navigational tasks, including appointment scheduling, billing inquiries, and hospital information access.

### Data Collection

Retrospective interaction logs: We extracted 20,179 authentic interaction logs generated between June and December 2025. The extracted variables included the interaction timestamp, agent type, user sex, age, and query content.

Cross-sectional survey: In December 2025, an electronic survey was randomly distributed to users via the “Doctor Xiao Yi” platform. A total of 152 valid responses were collected. The instrument assessed demographic characteristics, usage motivations, satisfaction scores, and behavioral changes in healthcare seeking (Table 1).

**Table 1:** Responses to questions Q1–Q5 on the use of the AI health assistant “Doctor Xiao Yi” (n = 152)

Question	Response option	n (%)
Q1. Main reasons for consulting the AI assistant during out-of-hours (OOH)^a^	A. Sudden physical discomfort or anxiety with no immediate access to a physician	84 (55.3)
	B. Sleep disturbance or waking up at night with unresolved medication or dietary concerns	48 (31.6)
	C. Avoid disturbing physicians; AI consultation perceived as more convenient	92 (60.5)
	D. Busy daytime schedule, only able to focus on health issues at night	34 (22.4)
Q2. Reasons for frequently consulting the AI assistant about post-transplant diet or physical activity^a^	A. Online information is inconsistent; preference for hospital-authorized AI advice	111 (73.0)
	B. Discharge education was provided but forgotten or not clearly remembered	76 (50.0)
	C. Physicians mainly emphasized medication management, with limited lifestyle guidance	63 (41.4)
	D. Disagreement among family members, requiring authoritative confirmation	41 (27.0)
Q3. After receiving advice from the AI assistant, would you still visit a hospital for the same issue	A. Depends on the situation; would visit if AI recommends hospital care	93 (61.2)
	B. No, the issue was resolved without further medical consultation	38 (25.0)
	C. Yes, AI advice is considered reference only; physician confirmation is required	21 (13.8)
Q4. Overall satisfaction score for the AI assistant (maximum 10 points)	9–10 points	114 (75.0)
	8 points	21 (13.8)
	≤7 points or unclear response	17 (11.2)
Q5. Willingness to recommend the AI assistant to other transplant recipients	Yes	145 (95.4)
	No	7 (4.6)

^a^Q1 and Q2 were multiple-choice questions; percentages were calculated based on the total number of respondents (n = 152). Therefore, the percentages may exceed 100%.

### Data Categorization and Semantic Processing

Conversation logs were categorized using a hybrid approach of keyword matching and expert manual validation. Interactions were classified into six distinct domains: medication and rejection, diet and lifestyle, symptoms and infection, test results, appointment, and payment. The temporal distribution was mapped on a 24-h cycle to identify usage peaks.

### Statistical Analysis

Data cleaning, preprocessing, and statistical analyses were performed using Python (v3.10), leveraging the Pandas (v1.5.3) and SciPy (v1.10.1) libraries.

Descriptive statistics: Continuous variables with a normal distribution are expressed as the mean ± standard deviation (SD), whereas nonnormal variables (e.g., age) are presented as the median (interquartile range [IQR]). Categorical variables are reported as frequencies (n) and percentages (%).

Hypothesis testing: Differences in age distribution between user groups were assessed using the Mann‒Whitney *U* test because of skewed data distribution. The Pearson Chi-square test was used to compare sex composition and the distribution of inquiry topics. To evaluate the “sentinel effect” at specific time points (e.g., 4:00), a two-sample *Z* test for proportions was applied to compare normalized interaction frequencies.

Significance: All tests were two-tailed, with statistical significance defined as *P* < 0.05.

## Results

### Demographic Characteristics and Temporal Patterns: The “Nocturnal Sentinel” Effect

**Fig. 1:**
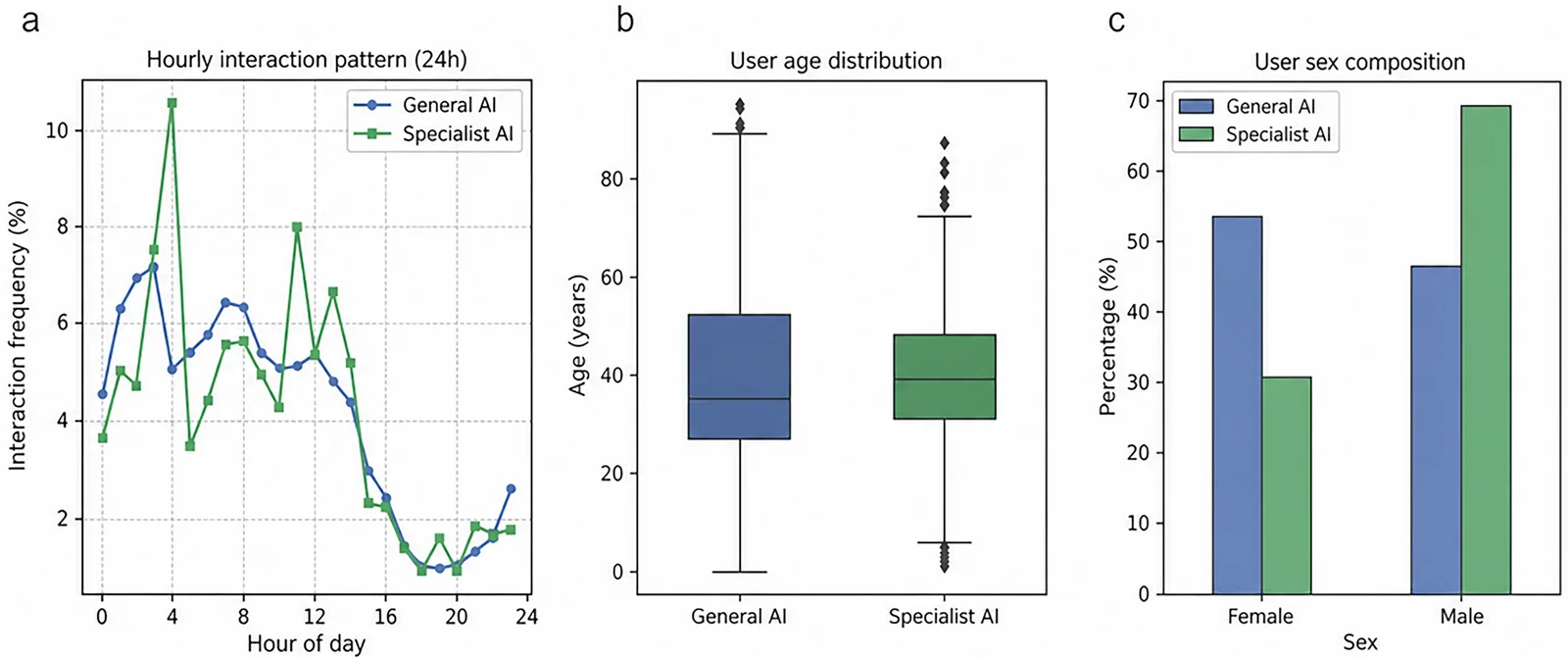
Comparison of user demographics and temporal usage patterns (n = 20,176). **a** Hourly interaction frequency (normalized). The specialist AI shows a statistically significant peak at 4:00 AM (*P* < 0.001, *Z* test), indicating specific nocturnal demand. **b** Age distribution. The users of specialist AI were significantly younger than those of general AI (*P* < 0.001, Mann‒Whitney *U* test). **c** Gender composition. Compared with general AI, specialist AI was associated with a significantly greater proportion of male users (*P* < 0.001, Chi-square test).

To evaluate the effectiveness of domain-specialized artificial intelligence in transplant care, we compared a general AI agent (Nan Xiao Yi) used in our hospital with our custom-built specialist AI agent (Doctor Xiao Yi) for transplant recipients. A total of 20,176 valid interaction logs were analyzed. The Nan Xiao Yi handled most of the volume (18,888 interactions, 93.6%), while the Doctor Xiao Yi managed 1,288 interactions (6.4%). Despite the disparity in volume, the two agents served distinct user populations with significantly divergent usage patterns (Fig. 1).

Demographic divergence: The user base for Doctor Xiao Yi was significantly younger than that of Nan Xiao Yi (*P* < 0.001, Mann‒Whitney *U* test), with a median age of approximately 40 years compared to 50 years for the general agent. This demographic (30–50 years) aligns with the active rehabilitation phase of transplant recipients. Furthermore, the sex distribution for Doctor Xiao Yi was predominantly male (>60%, *P* < 0.001; Pearson’s Chi-square test), a pattern highly consistent with the epidemiological sex distribution of organ transplant recipients, indicating precise engagement with the target clinical population.

Temporal divergence: The activity curves revealed a striking contrast in accessibility. Nan Xiao Yi exhibited a typical “outpatient pattern”, with activity peaking during standard business hours (08:00–17:00). In contrast, Doctor Xiao Yi demonstrated a distinct “Nocturnal Sentinel” effect. Statistical analysis revealed a significant usage peak at 04:00 AM (*P* < 0.001, two-sample *Z* test), where the interaction frequency accounted for approximately 3.0% of its total daily volume—nearly 15 times higher than that of the general agent (0.2%). This specific nocturnal activity unmasks a critical, previously underserved demand for medical support during deep night hours.

### Functional Positioning: Administrative Gateway vs. Digital Health Companion

**Fig. 2:**
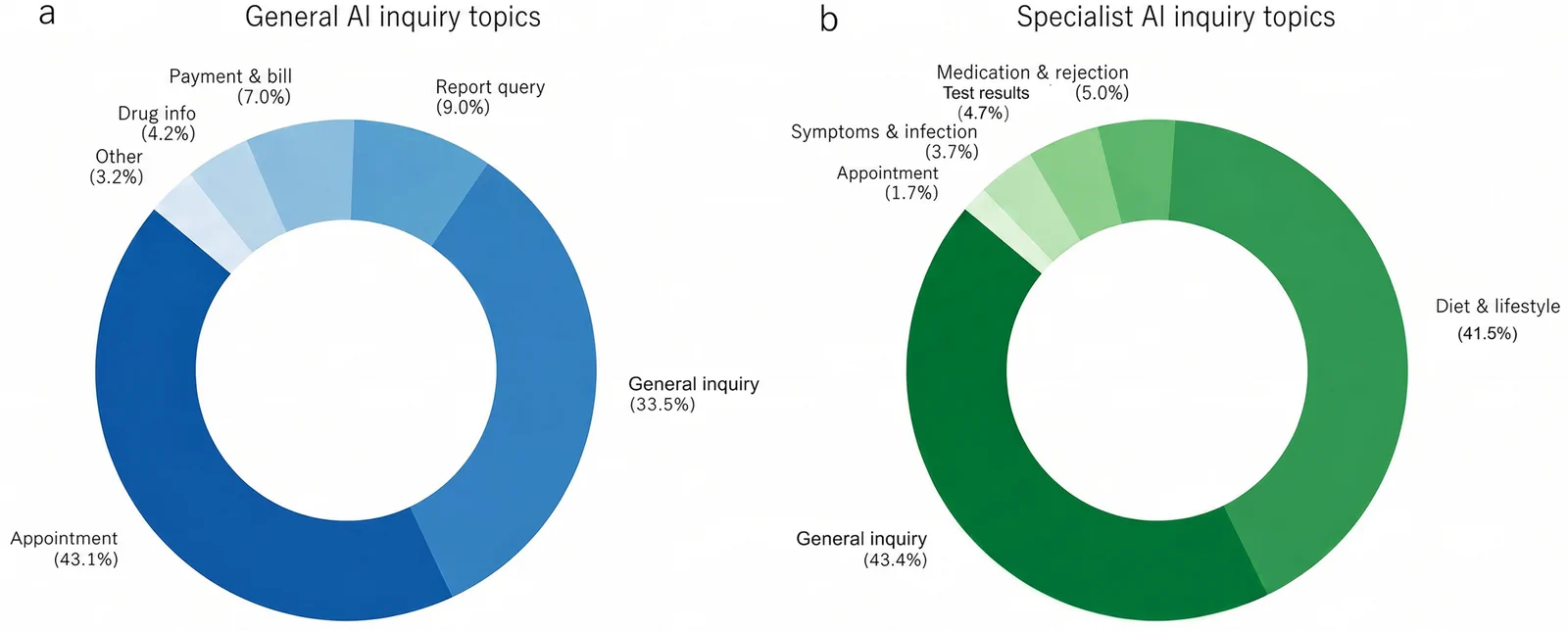
Comparative analysis of inquiry topics and functional positioning between general and specialist AI agents (n = 20,176). The pie charts illustrate the overall distribution of user intent over a 24-h period, highlighting the divergent roles of the two agents. **a** Inquiry distribution for the general AI (n = 18,888). Interactions are predominantly driven by appointments (43.1%) and general inquiries (33.5%), typically concerning hospital logistics. This pattern characterizes general AI as an administrative gateway for facilitating hospital access and workflow efficiency. **b** Inquiry distribution for specialist AI (n = 1,288). The profile reveals a strong clinical and lifestyle focus, with general inquiries (43.4%) and diet and lifestyle (41.5%) constituting most interactions. Combined with specific queries on medication (5.0%) and test results (4.7%), this distribution underscores the specialist AI’s role as a “digital health companion” supporting daily self-management and quality of life for transplant recipients. The “general inquiry” category for the general AI agent predominantly comprises queries regarding hospital operational hours and departmental triage (e.g., “Which department for headache”), and administrative logistics (e.g., invoices, location), reflecting its role as a digital front desk. The “general support” category for the specialist AI agent primarily includes preoperative consultation requests (e.g., surgery preparation), administrative inquiries (e.g., doctor referrals), and long-tail symptom descriptions not captured by standard keyword filtering.

Analysis of inquiry topics over the 24-h cycle revealed two fundamentally different service roles (Fig. 2):

(1) Nan Xiao Yi as an “Administrative Gateway”: Interactions were dominated by “appointments” (43.1%) and “general inquiries” (33.5%). Qualitative analysis confirmed that users primarily utilized Nan Xiao Yi to enhance logistical efficiency, such as by securing appointment slots and navigating hospital geography.

(2) Doctor Xiao Yi as a “Digital Health Companion”: The inquiry profile shifted sharply toward clinical and lifestyle management. “diet & lifestyle” queries constituted 41.5% of interactions, equaling the volume of “general inquiries” (43.4%). Furthermore, specific clinical categories such as “medication” (5.0%) and “test results” (4.7%) were significantly more prevalent. These findings suggest that patients perceive Doctor Xiao Yi not only as a tool but also as a professional partner essential for self-management.

### Bridging the Care Gap: Analysis of OOH Service

**Fig. 3:**
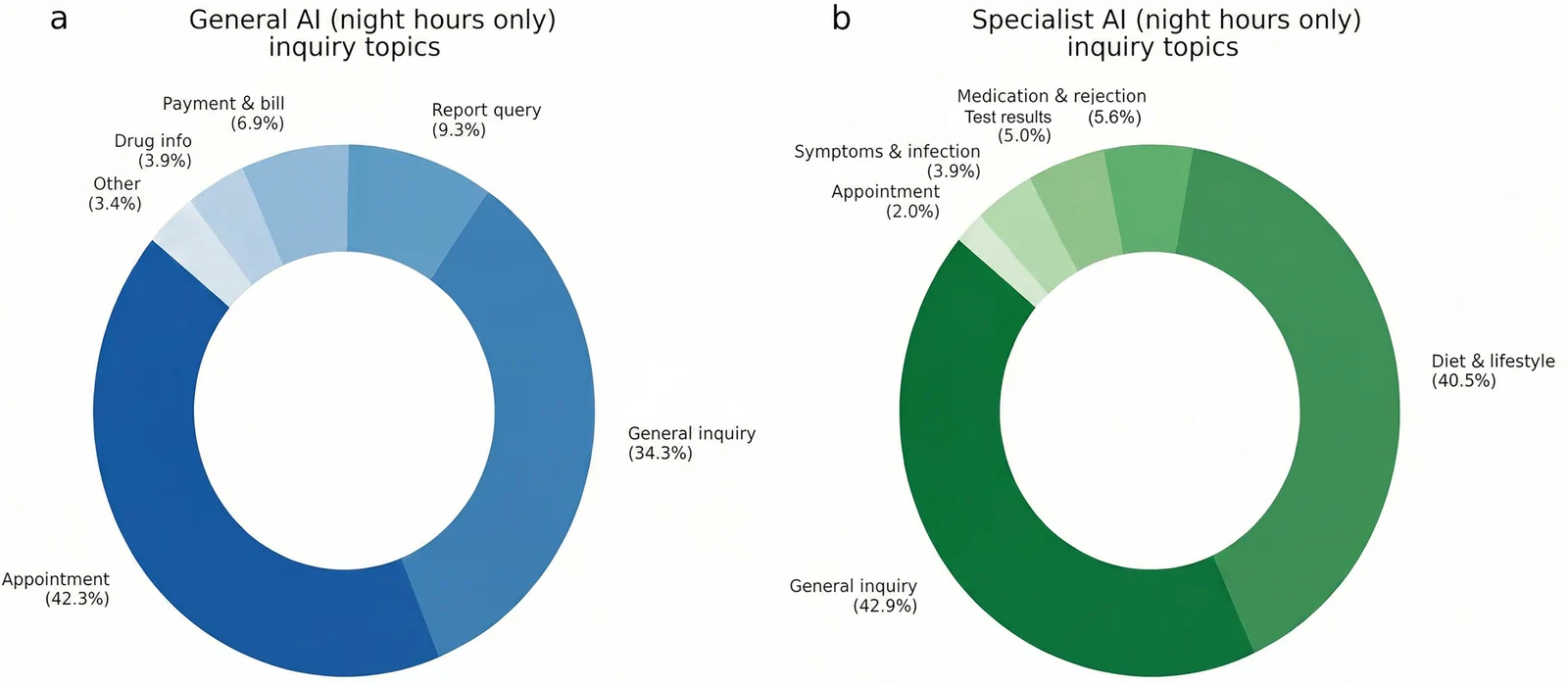
Comparative analysis of inquiry patterns between general and specialist AI agents during OOH (18:00–8:00). The charts illustrate the divergence in user intent during hours when human medical staff are largely unavailable. **a** Inquiry distribution for general AI (n = 10,665). Interactions are predominantly driven by appointments (42.3%), indicating that patients utilize general AI primarily as a logistical tool to secure medical resources for the following day. **b** Inquiry distribution for specialist AI (n = 694). The usage is dominated by general inquiries (42.9%) and diet & lifestyle (40.5%). This pattern suggests that the specialist AI functions as a “digital night-time guardian,” providing immediate guidance on postoperative care and symptom triage. The category “general inquiry” differs contextually between agents: for general AI, it refers to logistical queries (e.g., hospital contacts, locations); for specialist AI, it refers to context-specific medical or recovery questions (e.g., symptom management, rehabilitation readiness).

Focusing specifically on OOH (18:00–8:00), the divergence in service logic became even more pronounced (Fig. 3):

Nan Xiao Yi: Even during OOH periods, the primary intent remained logistical (“appointments”: 42.3%), reflecting patients’ need to plan their healthcare itinerary for the following day.

Doctor Xiao Yi: OOH usage was characterized by a surge in “general inquiries” (42.9%) and “diet & lifestyle” advice (40.5%). A qualitative breakdown of these “general inquiries” revealed a high prevalence of long-tail clinical questions (e.g., “postoperative fever management” and “exercise timing”) rather than administrative logistics. Notably, during the deep night window (22:00–6:00), the prevalence of “dietary contraindications” and “acute symptoms” (e.g., fever and wound discomfort) increased to 52.4%. These findings confirm that Doctor Xiao Yi effectively fills the medical service vacuum when human staff are unavailable.

### User Experience and Motivational Drivers

**Fig. 4:**
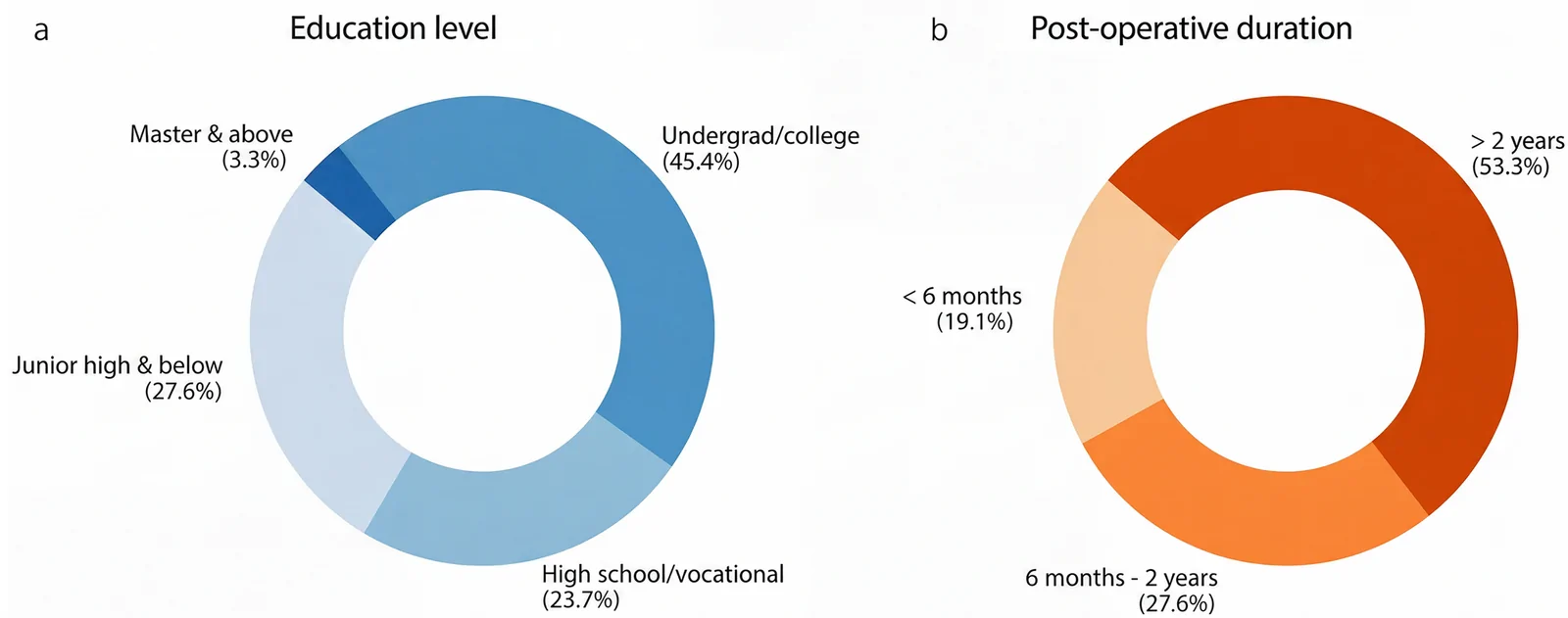
Demographic characteristics of the survey respondents (n = 152). **a** Distribution of education levels. The cohort demonstrated relatively high health literacy, with 45.4% of the respondents holding an undergraduate degree (undergrad/college). **b** Distribution of postoperative duration. Most users (53.3%) were long-term survivors (>2 years), indicating that a stable patient population focused on long-term home management.

**Fig. 5:**
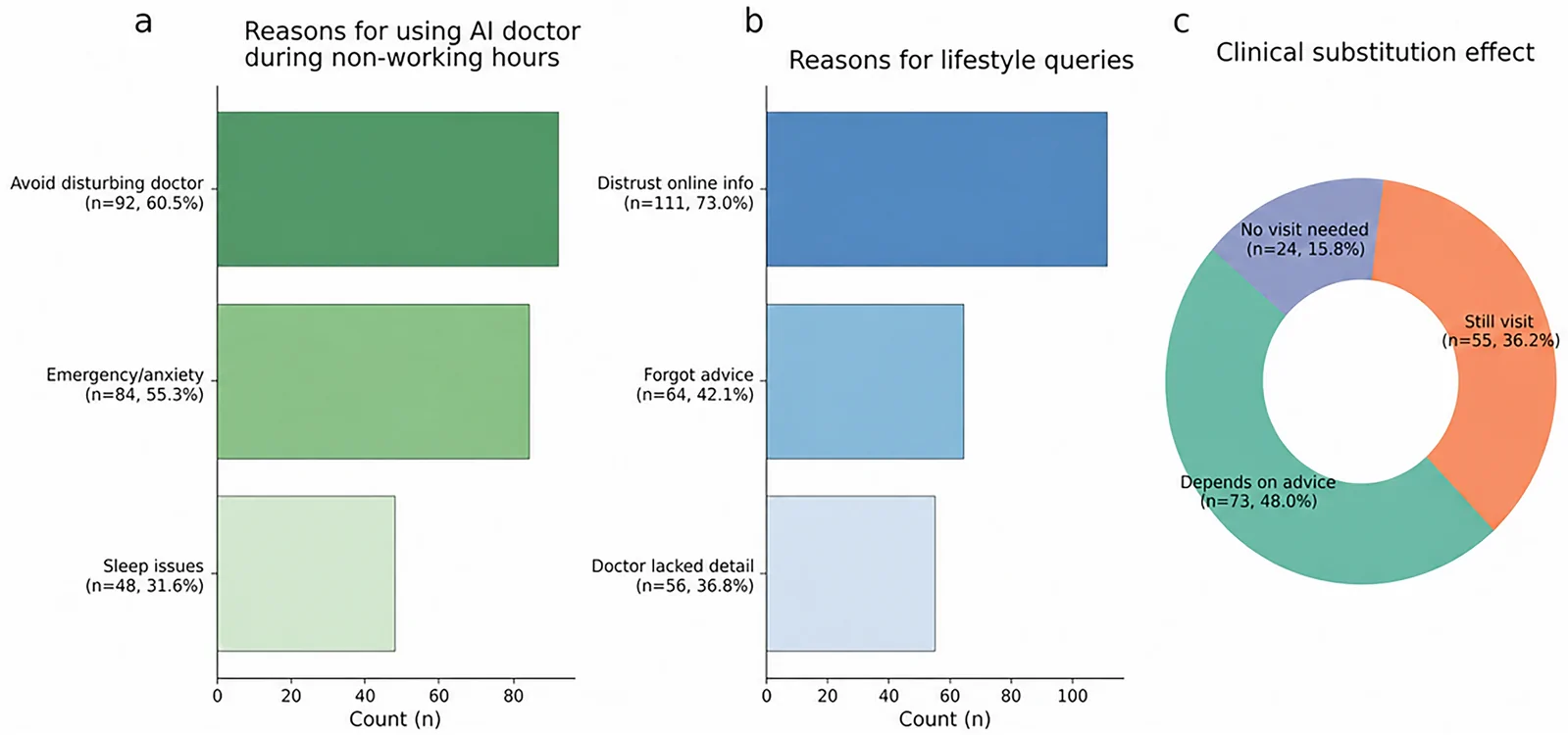
Analysis of motivational drivers and clinical substitution effect (n = 152). **a** Reasons for using an AI doctor during OOH. “Avoid disturbing doctors” and “emergency/anxiety” appear to be the most prominent drivers, highlighting the dual role of the agent in reducing social pressure and addressing urgent needs. **b** Motivations for consulting on lifestyle issues. The visualization highlights that “doctor lacked detail” (insufficient information from doctors) and “distrust online info” (skepticism of online information) are the primary factors leading patients to seek AI assistance. **c** Clinical substitution effect. Most patients showed rational triage behavior, with 48.0% deciding their hospital visit based on AI advice (“depends on advice”) and 15.8% resolving their issues without a visit (“no visit needed”).

A cross-sectional survey of 152 transplant recipients (Table 1 and Figs. 4, 5) provided insight into the psychological drivers behind these behavioral patterns:

Respondent profile: The cohort comprised predominantly long-term survivors (>2 years post-transplant: 53.3%) with a relatively high level of health literacy (high school education or above: 72.4%). The mean satisfaction score was 8.7/10, indicating high acceptance.

Psychological motivations: When asked why they utilized the AI during OOH, 60.5% cited “avoiding disturbing the doctor” as the primary reason, followed by “anxiety from sudden discomfort” (55.3%). Doctor Xiao Yi is identified as a “psychological safety valve”, mitigating the social pressure patients feel about contacting clinicians outside of office hours.

Trust and information seeking: For lifestyle inquiries, “distrust of online information” was the overwhelming driver (73.0%), highlighting the value of an authoritative, hospital-endorsed knowledge base.

Clinical substitution effect: In terms of healthcare utilization, 63.8% of the respondents indicated that the AI influenced their decision to visit the hospital (48.0% “depends on advice”, 15.8% “no visit needed”). These findings suggest that AI significantly influences patients’ self-reported behavioral intentions regarding healthcare seeking. While this indicates a substantial potential for prehospital triage, it is important to note that these represent intended actions rather than measured reductions in actual emergency visits.

## Discussion

Patients with multimorbidity and polypharmacy are more likely to make frequent use of OOH medical services [3]. Organ transplant recipients, inherently defined by these complexities, exhibit rigid medical demands during nights and holidays. Our study, through a mixed-methods approach, not only validated the “hidden demands” of this population but also elucidated the unique role of vertical LLMs in filling the “spatiotemporal” and “emotional” vacuums of postoperative care.

### The “Sentinel” at 4:00 AM: Dual Support for Symptoms and Affect

“Authority dependence” refers to the overreliance on authoritative sources or judgments, leading to insufficient independent reasoning and decision-making. Research has indicated that patients face significant hesitation stemming from both physical barriers and the psychological internalization of medical power structures. Patients, particularly elderly patients, often engage in excessive self-censorship, fearing that their help-seeking behavior might be deemed “inappropriate” or “noncompliant” by clinicians. This fear of being judged for trivial concerns leads to silence and decision paralysis [3, 7]. In our study, while both Nan Xiao Yi (general AI) and Doctor Xiao Yi (specialist AI) were used during OOH periods, the latter demonstrated a distinct “Nocturnal Sentinel” effect, highlighted by an anomalous activity peak at 4:00 AM. Our survey revealed that 60.5% of users accessed the AI during these hours, driven by a “reluctance to disturb the doctor”. These findings align with the literature suggesting that chronic patients often suppress OOH inquiries because of “authority dependence”.

Recent published studies suggest that compared with human physicians, AI-based chatbots may demonstrate higher levels of perceived empathy and improved readability in certain clinical communication scenarios. The “nonhuman” nature of AI reduces social pressure, encouraging patients to disclose sensitive issues (e.g., sexual dysfunction or drug side effects) that they might withhold from human providers [8]. Transplant recipients are further burdened by neuropsychiatric sequelae, including anxiety and sleep disorders. Nocturnal symptoms such as blood pressure fluctuations, while not emergencies, amplify this distress. The clinical significance of this 4:00 AM peak aligns with that of physiological circadian nadirs and potential fluctuations in immunosuppressant blood concentrations, compounded by the psychological vulnerability and isolation typical of deep night hours. In this context, Doctor Xiao Yi serves not only as an information source but also as an emotional buffer. However, whether this digital buffering translates to a reduction in actual adverse clinical events requires future objective evaluation.

### Technological Validity: Rebuilding Trust Via the “Dual-Source Knowledge Base + GraphRAG” Architecture

Despite their prowess in general exams, general LLMs (e.g., ChatGPT and Gemini) are prone to “hallucinations” and the fabrication of references in complex clinical scenarios such as stem cell transplantation [5, 6]. Koller et al. demonstrated that compared with simple prompt engineering, employing advanced RAG significantly enhances response consistency [9]. Furthermore, adopting a construction philosophy like Med-Pal—anchoring the model in high-quality medical textbooks and guidelines rather than uncurated pretraining data—provides the “evidence-based constraint” essential for patient trust [9, 10]. A network meta-analysis by Wang et al. further corroborated that models fine-tuned for specific tasks consistently outperform generalist models in domain-specific accuracy [11]. Therefore, combining the RAG score with an independent knowledge base is an effective method for eliminating LLM hallucinations. Our data revealed that 73% of patients relied on specialist AI because of distrust of fragmented online information, validating our “Dual-Source Knowledge Base + GraphRAG” architecture.

### Safety Boundary

AI as “copilot," not “captain”: Despite high patient trust, the decision-making capacity of AI must be viewed dialectically. While LLMs excel in health education and basic triage, studies indicate that they still lag behind human experts in managing complex postoperative complications (e.g., distinguishing anastomotic leaks from rejection) and may provide incomplete treatment plans [6, 12]. For instance, ChatGPT-4 has been shown to occasionally underestimate the severity of emergent conditions [12]. Therefore, the MLRDN implemented by Doctor Xiao Yi is critical. As emphasized in the Med-Pal study, strict “guard-railing” must be enforced to handle adversarial prompts and high-risk keywords [10]. We posit that the role of the AI is strictly defined as a “clinical copilot”—responsible for information gathering and preliminary guidance—while the final clinical authority, especially regarding medication adjustment and emergency transfer, remains with the human physician.

### Potential in Optimizing Medical Resource Allocation

High volumes of repetitive, nonurgent inquiries significantly strain outpatient resources, a prevalent issue in multimorbidity management [13]. Effectively filtering these demands to prioritize critical cases is a core challenge for hospital administration. Our interaction logs confirm that most Doctor Xiao Yi’s inquiries are related to diet and rehabilitation. With 63.8% of the respondents expressing a behavioral intention to rely on AI for initial guidance, the system shows potential as a preliminary prehospital triage tool. However, the actual interception of noncore inquiries and the subsequent redistribution of scarce medical resources to critically ill patients remain hypothetical in this descriptive study and must be validated through future analyses of hospital admission records.

### Limitations and Ethical Challenges

This study has several limitations. First, the survey sample (n = 152) was collected via random pop-ups among active users, which inherently introduces selection bias, as respondents are likely to possess greater digital literacy and engagement. Consequently, the “63.8% interception rate” reflects subjective behavioral intention rather than actual triage, requiring future validation through objective outcome tracking, such as evaluating unplanned readmission rates and OOH emergency department visits. Second, the “digital divide” remains a concern; as noted by Laymouna et al., chatbots may present usage barriers for populations with lower technical literacy, such as elderly individuals, potentially excluding a segment of “silent” transplant recipients [14]. Third, despite anonymization, the depth of interaction involves sensitive data. Balancing cloud-based inference with local data privacy remains a significant legal bottleneck [10, 15]. Finally, a legal void exists regarding liability attribution in cases where AI-provided lifestyle advice indirectly leads to adverse outcomes [14].

## Conclusions

This study provides the first real-world evidence confirming the feasibility and high patient acceptance of specialist AI agents in the domain of organ transplantation. Technologically, the “dual-source knowledge base + graphRAG+ multiagent framework” architecture effectively mitigates the “hallucination” risks inherent in generalist models, establishing a trust mechanism essential for post-transplant care. We identified a distinct “Nocturnal Sentinel” effect, where the specialist agent (Doctor Xiao Yi) successfully connects the “temporal blind spots” of traditional healthcare. Unlike the general AI agent (Nan Xiao Yi), which functions primarily as an administrative assistant, the specialist agent acts as a “digital health copilot”, offering critical physiological and psychological buffering when human resources are inaccessible. Nevertheless, future prospective studies with objective clinical endpoints are needed to evaluate its true impact on actual healthcare utilization and long-term medical effectiveness.

## Supplementary Information

Below is the link to the electronic supplementary material.

Supplementary file1 (DOCX 1776 KB)

## Data Availability

The data that support the findings of this study are available from the corresponding author upon reasonable request.
