# Supplementary material for: Real-World Analysis of Organ Transplantation-Specific Agent Based on Large Language Model in Post-Transplant Self-Management During Off-Hours: A Mixed-Methods Study
Source: Curr Med Sci. 2026 Jul 1;46(4):1004–13. doi: 10.1007/s11596-026-00219-3 (PMC13503416; doi:10.1007/s11596-026-00219-3)
Supplement: Supplementary file 1 [file 11596_2026_219_MOESM1_ESM.docx]

**Specialist AI “Circuit-Breaker Mechanism” Positive and Negative Semantic Refinement Table**

It is recommended to upgrade the simple “vocabulary matching” approach to a semantic understanding model based on “symptom + severity/accompanying symptoms”. Six major critical systems have been listed:

● Positive Semantic Refinement Table for the Specialist AI “Circuit-Breaker Mechanism”.

1. **Vital Signs and Systemic Emergencies (Red Zone/Level 1).** These conditions typically indicate unstable vital signs, and the AI must immediately trigger the highest-level alarm, bypass follow-up questions, directly display a pop-up prompting the user to call 120, and transfer the session to human staff.

| Original term | AI semantic training expansion (including synonyms/colloquialisms/accompanying symptoms) | Exclusion/Differential logic (prompt optimization hints) |
| --- | --- | --- |
| Save me, dying, going to die  Coma, cannot be awakened | Feeling of impending doom, suffocating, and going to die, save me, I only have one breath left  Loss of consciousness, unresponsive to shouting, unresponsive to pushing, eyes rolling back, fainted, and didn’t wake up | Exclude exaggerated rhetoric (e.g., “tired to death”, “laughing to death”). Requires context and emotion analysis  Distinguished from natural sleep (“sleeping so deeply they can’t be woken up”) |
| Persistent high fever, chills | Temperature > 39 °C with shivering, antipyretics ineffective, febrile convulsion, whole body cold and shivering | Focus on identifying postoperative high fever (indicating severe infection/sepsis) |
| Severe burns | Skin charred, large-area scalds, chemical splash, skin peeled off | / |

2. **Cardiovascular and Cerebrovascular Systems (Extremely High Risk)**. This is a high-incidence area for misjudgment, and the AI must identify typical symptom descriptions.

| Original term | AI semantic training expansion | Exclusion/differential logic |
| --- | --- | --- |
| Cardiac arrest, no heartbeat | Cannot feel pulse, no chest movement, ashen face | / |
| Severe chest pain, crushing pain | Chest feels pressed by a stone, chest pain radiating to the back/left shoulder, chest pain with heavy sweating, severe chest tightness | Exclude minor twinges or pain unrelated to breathing. Focus on capturing three keywords: “crushing sensation,” “impending doom,” and “sweating” |
| Sudden collapse, convulsions | Epileptic seizure, foaming at the mouth, limbs stiff and twitching, sudden fall | / |
| Severe palpitations, irregular heart rate | Heart jumping into throat, pulse > 120 or < 40 bpm, heart beating wildly | If smart device data is available (e.g., Apple Watch indicates AFib), double the weighting factor |
| High blood pressure, systemic edema | Systolic BP ≥ 180 mmHg, Diastolic BP ≥ 110 mmHg, dizziness with nausea, pitting edema | Focus on chronic disease/hypertensive patients, and must be alert to hypertensive emergencies |

3. **Nervous System (Stroke Warning).** The emphasis here is on the word “sudden”; the AI must be highly sensitive to temporal adverbs.

| Original term | AI semantic training expansion | Exclusion/differential logic |
| --- | --- | --- |
| Unilateral limb weakness, slurred speech | Cannot hold chopsticks, walking leaning to one side, crooked mouth, thick tongue when speaking, drooling  (FAST principle) | Exclude post-dental anesthesia, chronic old hemiplegia (must identify “sudden” or “new onset”) |
| Severe headache | Feels like head will explode, thunderclap headache, worst headache of my life, headache with projectile vomiting | Exclude chronic migraines, tension headaches. Focus on “never had before” and “explosive sensation” |
| Blurred vision, blindness | Vision suddenly went pitch black, feels like a dark curtain covering, seeing double (diplopia) | Exclude effects after using eye drops, progressive changes from presbyopia |

4. **Digestive System and Acute Abdomen.** Focus on distinguishing common stomachaches from an acute abdomen.

| Original term | AI semantic training expansion | Exclusion/differential logic |
| --- | --- | --- |
| Massive hemorrhage, hematemesis, melena | Vomiting blood, mouthfuls of bright red blood, passing tarry stools, stool looks like asphalt, hematuria (urine like meat-washing water) | Exclude pseudo-melena caused by eating red dragon fruit/duck blood (AI can attempt to ask about diet, but if accompanied by dizziness, trigger circuit-breaker) |
| Board-like abdominal rigidity | The belly is as hard as a wooden board, cannot press down, screams in pain when touched (rebound tenderness), dare not move, dare not breathe | This is a sign of peritonitis, with an extremely high risk |
| Severe vomiting, inability to eat | Vomiting whatever is eaten, vomiting yellow fluid/bile, no passing of gas or stool (signs of intestinal obstruction), diarrhea (more than 5 times), so weak that they cannot stand, whole body has no energy | Exclude morning sickness (requires department context) |

5. **Obstetrics and Gynecology (Special Populations)**. For obstetric and postoperative gynecological patients, the alerting threshold needs to be lowered.

| Original term | AI semantic training expansion | Exclusion/differential logic |
| --- | --- | --- |
| Disappearance of fetal movement | Baby stopped moving, and I haven’t felt fetal movement all morning | Absolute obstetric emergency, no differentiation needed |
| Massive vaginal bleeding | Sanitary pad soaked instantly, blood running down legs, large blood clots | Distinguish from normal menstrual volume (e.g., “much more than usual menstruation”) |
| Abdominal pain with bleeding | Belly hurts and bleeding below (signs of ectopic pregnancy rupture/placental abruption) | / |

6. **Psychological Crisis and Social Safety (Legal Red Line).** These triggers involve not only medical care but also the legal obligation to report to authorities.

| Original term | AI semantic training expansion | Exclusion/differential logic |
| --- | --- | --- |
| Suicide, don’t want to live | Currently cutting wrists, took a bottle of pills, standing on the roof, life is meaningless, want to be free | Requires sentiment analysis. Exclude complaints (“work makes me want to kill myself”). If specific plans or tools are detected, immediately trigger the circuit breaker. |
| Violence, sexual assault | Beaten by husband, beaten, unable to move, forced to have relations, covered in bruises | Involves domestic violence/sexual assault. AI must activate the “Privacy Protection + Advice to Call Police” protocol. |

**Specialist AI “Critical Value Circuit-Breaker” Negative Sample Training Set**

1. **Emotional Venting and Exaggerated Rhetoric (Disaster Area).** Patients often use somatic words to describe psychological feelings or life stress, and the AI is most prone to misjudgment here.

| Trigger keyword | Negative sample corpus (user’s words) | AI intended judgment | Why NOT trigger the circuit-breaker |
| --- | --- | --- | --- |
| Die, don’t want to live | “It’s so hard to get a registration number this time, I’m worried to death.” | Expressing complaint/anxiety | Rhetorical device, no suicidal intent |
| Die, don’t want to live | “Taking this medicine every day, it tastes so bad I want to die.” | Expressing dissatisfaction with the treatment plan | Emotional venting, not a real suicide plan |
| Heartache, heart pain | “This surgery cost tens of thousands, my heart aches to death.” | Expressing financial pressure | The “heartache” here is a psychological feeling, not organic cardiac pain |
| Massive hemorrhage, vomiting blood | “I had a massive hemorrhage (spent a lot of money) to get this expert appointment.” | Expressing a huge expense | Slang, not medical bleeding |
| Headache, explosive | “The kid is so disobedient, my head is going to explode.” | Expressing annoyance | Non-pathological headache |
| Stop breathing, cannot catch breath | “I just ran to catch the bus, and I almost ran out of breath.” | Expressing fatigue after exercise | Physiological response, not pathological dyspnea (needs to be combined with whether it relieves quickly) |

2. **Stating Past History and Others’ Conditions.** The AI needs to distinguish between “happening right now” and “happened in the past” or “happened to someone else”.

| Trigger keyword | Negative sample corpus (user’s words) | AI intended judgment | Why NOT trigger the circuit-breaker |
| --- | --- | --- | --- |
| Coma, unconscious | “My father was in a coma for a month ten years ago due to a car accident.” | Stating past family history | Time adverb indicates “past”, not a current emergency |
| Massive hemorrhage | “Doctor, I had a massive hemorrhage when I gave birth to my first child. Will it affect this time?” | Consulting on delivery risk | Describing obstetric past history to assess risk, not current bleeding |
| Heart attack, sudden death | “Just saw on the news a celebrity died of a heart attack, I’m a bit worried.” | Expressing anxiety/consulting prevention | Describing a third-party event, not the user’s own symptoms |
| Suicide | “My neighbor committed suicide by drinking pesticide; the smell was awful.” | Chitchat/describing environment | Describing third-party behavior |

3. **Negative Sentences and Symptom Relief Keywords** appear, but are preceded by “no”, “not”, or indicate “getting better”.

| Trigger keyword | Negative sample corpus (user’s words) | AI intended judgment | Why NOT trigger the circuit-breaker |
| --- | --- | --- | --- |
| Severe abdominal pain | “Took the medicine you prescribed, now I no longer have severe abdominal pain.” | Feedback on treatment effect (improvement) | Contains keyword, but the logic is “negative/termination” |
| Fever, high fever | “I don’t have a fever now, just a runny nose.” | Stating the current condition | Negative sentence |
| Chest pain | “I have no chest pain, only a little chest tightness.” | Differential diagnosis description | Explicitly rules out critical symptoms |

4. **Inquiries about Drug Side Effects and Hypothetical Questions.** Panic inquiries after patients read drug instructions or search online.

| Trigger keyword | Negative sample corpus (user’s words) | AI intended judgment | Why NOT trigger the circuit-breaker |
| --- | --- | --- | --- |
| Sudden death, cardiac arrest | “Will taking this medicine cause cardiac arrest?” | Consulting drug side effects | Hypothetical inquiry, not a symptom description |
| Blindness | “If I don’t have the surgery, will I go blind in the future?” | Consulting disease prognosis | Asking about future possibilities |
| Massive hemorrhage | “What should I do in case of a massive hemorrhage after going home?” | Consulting the emergency plan | Has not happened, seeking guidance |

5. **Specialty-Specific Normal Manifestations (Requires Department Context)**. Terms that look alarming to ordinary people may be normal after surgery in specific departments.

| Trigger keyword | Negative sample corpus (user’s words) | AI intended judgment | Why NOT trigger the circuit-breaker |
| --- | --- | --- | --- |
| Hematuria | “Doctor, just had a cystoscopy. Is a little blood in the urine normal?” | Postoperative consultation | Transient hematuria after a urological examination is a common phenomenon (requires defining a threshold: e.g., only trigger if “bright red and has blood clots”) |
| Pus discharge | “There’s a little yellow secretion (referred to as pus by the patient) at the drainage tube site.” | Wound care consultation | May be fat liquefaction or normal exudation; it should guide the patient to take a photo for confirmation rather than directly triggering the circuit-breaker |
